# Understanding why people participate in HIV surveillance

**DOI:** 10.2471/BLT.14.135756

**Published:** 2015-05-01

**Authors:** Basia Zaba, Georges Reniers, Emma Slaymaker, Jim Todd, Judith Glynn, Amelia Crampin, Mark Urassa, Tom Lutalo, Marie-Louise Newell, Victoria Hosegood, Samuel Clark, Simon Gregson

**Affiliations:** a London School of Hygiene & Tropical Medicine, Keppel Street, London, WC1E 7HT, England.; b Karonga Prevention Study, Chilumba, Malawi.; c National Institute of Medical Research, Mwanza, United Republic of Tanzania.; d Rakai Health Sciences Program, Rakai, Uganda.; e Southampton University, Southampton, England.; f Africa Centre for Health and Population Studies, Mtubatuba, South Africa.; g University of Washington, Seattle, United States of America.; h Imperial College, London, England.

People have argued that the benefits of human immunodeficiency virus (HIV) testing and counselling are so important that participants in HIV surveys must be given their HIV test results and that individuals who decline to receive their test results should be excluded from participation in such surveys.^1^^–^^3^

In early attempts at HIV surveillance, there were many logistical issues that complicated the return of test results and few advantages to infected individuals in receiving their test results.^4^ Now the situation has changed radically with the widespread roll-out of HIV treatment and care – which not only prolongs life but also reduces sexual and vertical transmission of HIV.^2^ We agree that researchers now have an obligation to offer and encourage post-test counselling as part of a research encounter but argue that there are both practical and ethical reasons to allow study participants to opt out of post-test counselling.

In African populations with HIV prevalence above 4%, between 30% and 81% of infected men and women have ever been tested for HIV.^5^ Those who know they are HIV-positive may be willing to donate blood samples for research purposes but may not want to repeat pre- and post-test counselling. The same may be true of those who feel certain that they are uninfected. Sexually active men and women are encouraged to be tested regularly. Research studies should provide the option for those who arrange to be frequently retested – so-called repeat testers – to be given their test results in a short format and be allowed to opt out of post-test counselling. They should also be prepared for participants who do not wish to collect their test results in any format. All participants – including those who provide blood samples for research but opt out of post-test counselling – can be asked to report their testing histories and testing motives.

Importantly, the opt-out provisions we discuss here align with the general principles of research ethics. We agree that there is public health utility in informing people of their HIV status but we think that this should not override the ethics of respect for individual autonomy. Informed consent procedures typically tell willing subjects that they have a right not to answer questions that make them uncomfortable and a right to withdraw from the study at any time without completing all the activities and procedures. Declining to receive a test result is an example of the right of participants to opt out of part of a study. Whether in research or clinical practice, public health utility would be better served through understanding peoples’ reasons for not wanting to receive an HIV test result – while informing individuals about all of the available testing and counselling options.

Rather than excluding those who decline post-test counselling, we have the obligation to understand the reasons for research participation – and non-participation – better, including attitudes to learning or confirming one’s HIV status. Longitudinal studies with repeated HIV testing are particularly well placed to investigate decision-making around HIV testing. The results of such studies could demonstrate whether refusal to participate or receive test results lead to biased estimates of HIV prevalence that weaken the public health utility of the data.^6^^,^^7^ They could also teach us about why and how people become motivated to be tested^8^ and that knowledge could lead to improvements in the design of programmes of HIV testing.
